# Neural mechanisms underlying rule selection based on response evaluation: a near-infrared spectroscopy study

**DOI:** 10.1038/s41598-022-25185-3

**Published:** 2022-11-30

**Authors:** Taeko Harada, Toshiki Iwabuchi, Atsushi Senju, Chikako Nakayasu, Ryuji Nakahara, Kenji J Tsuchiya, Yoko Hoshi

**Affiliations:** 1 Research Center for Child Mental Development, Hamamatsu University School of Medicine, Japan, 1-20-1 Handayama, Higashi-Ku, Hamamatsu, Shizuoka 431-3192 Japan; 2 United Graduate School of Child Development, Hamamatsu University School of Medicine, 1-20-1 Handayama, Higashi-Ku, Hamamatsu, Shizuoka 431-3192 Japan; 3 Early Childhood Education, Okazaki Women’s Junior College, 1-8-4 Nakamachi, Okazaki, Aichi 444-0015 Japan; 4 Department of Biomedical Optics, Hamamatsu University School of Medicine, 1-20-1 Handayama, Higashi-Ku, Hamamatsu, Shizuoka 431-3192 Japan

**Keywords:** Neuroscience, Cognitive neuroscience, Cognitive control

## Abstract

The ability of humans to use rules for organizing action demands a high level of executive control. Situational complexity mediates rule selection, from the adoption of a given rule to the selection of complex rules to achieve an appropriate response. Several rules have been proposed to be superordinate to human behavior in a cognitive hierarchy and mediated by different brain regions. In the present study, using a novel rule-selection task based on pre-response evaluations that require several cognitive operations, we examined whether the task is mediated by a specific region of the prefrontal cortex using near-infrared spectroscopy. We showed that the selection of rules, including prior evaluation of a stimulus, activates broader areas of the prefrontal and premotor regions than response selection based on a given rule. The results are discussed in terms of hierarchical cognitive models, the functional specialization of multiple-cognitive operations in the prefrontal cortex, and their contribution to a novel cognitive task.

## Introduction

Many human behaviors are rule-based, where a set of rules determines the action. For most daily tasks, we use not only simple rules that link an action to a specific stimulus (e.g., if the doorbell rings, we open the door) but also complex rules, such as choosing rules flexibly according to ongoing conditions. In particular, rule selection in daily life involves the consideration of possible responses in advance of the action, followed by deciding on the final action according to the evaluation of the situational appropriateness of the response. For example, if we were driving and an object suddenly falls in front of the car, we would choose to stop the car. However, if there is a car behind us, we would choose another response, such as making a detour, because suddenly braking may cause the car behind to collide with our car. These decisions are made after considering in advance what action would be most appropriate for the possible responses and consequences. In the above example, further higher-level rule adjustments are required to determine the appropriateness of the rule (i.e., whether the rule of “stop because of the falling objects,” the rule of “do not stop because a car is behind us,” or an entirely different response is more appropriate for the current situation). Such rule selection requires the coordination of various cognitive operations and is thus executed by the higher-level cognitive system. Therefore, such cognitive manipulation is considered a superordinate control system that operates within a cognitive hierarchy, which may be mediated by higher-order brain regions^1^. However, it is unclear whether a specific region is responsible for the integrative function as the central role of cognitive operation.

Previous studies have elucidated that the prefrontal cortex (PFC) of humans and non-human primates is responsible for the rule-based control systems involved in coordinating multiple cognitive processes that are essential for rule-based strategies. These processes include attentional set formation, rule encoding, and feedback integration, with links to motor responses in the premotor cortex (PMC)^2,3^, such as the encoding of behavioral responses^4–7^. Recent studies have focused on the functional organization of the human control system within the lateral PFC underlying hierarchical cognitive theory. In particular, multiple rule-based tasks, such as multitask paradigms, have been shown to involve the frontopolar cortex (FPC) as the apex of the hierarchical functional subdivision of the PFC along the rostral-caudal axis (i.e., gradient theories)^8–10^. Gradient theories state that higher-level rules are represented in progressively more rostral PFC regions along a rostral-to-caudal gradient^10–15^; this has been supported by several previous studies^9,16,17^. Additionally, recent evidence has suggested the existence of a hierarchical organization in the mid-dorsolateral region along the dorsal–ventral axis rather than in the FPC along the rostral–caudal axis^18–21^. In particular, the dorsolateral prefrontal cortex (DLPFC) has been implicated when the appropriate behavioral response is not a simple reaction to a stimulus but rather requires consideration of the current task set^10,17,22^. Furthermore, it has been suggested that the DLPFC plays a cortical integrator role, whereby it combines stimulus- and context-related information with temporal information about the task to ensure successful task performance^20^. Thus, the DLPFC appears to have a central function in coordinating and integrating multiple information processes along the dorsal–ventral axis. Based on these theories, we predicted that the rule-selection process responsible for the selection of appropriate behavior would be specifically engaged by the FPC or the DLPFC in the PFC as a superordinate control system for integrative manipulation in multiple information processes. However, it remains unknown which neural mechanisms underpin the selection of appropriate behavior based on prior evaluation of the consequences of a given rule-response. Moreover, there are currently no established tasks for assessing higher cognitive function through multiple cognitive operations that demand rule selection based on the consequences of rule-response evaluation.

**Figure 1:**
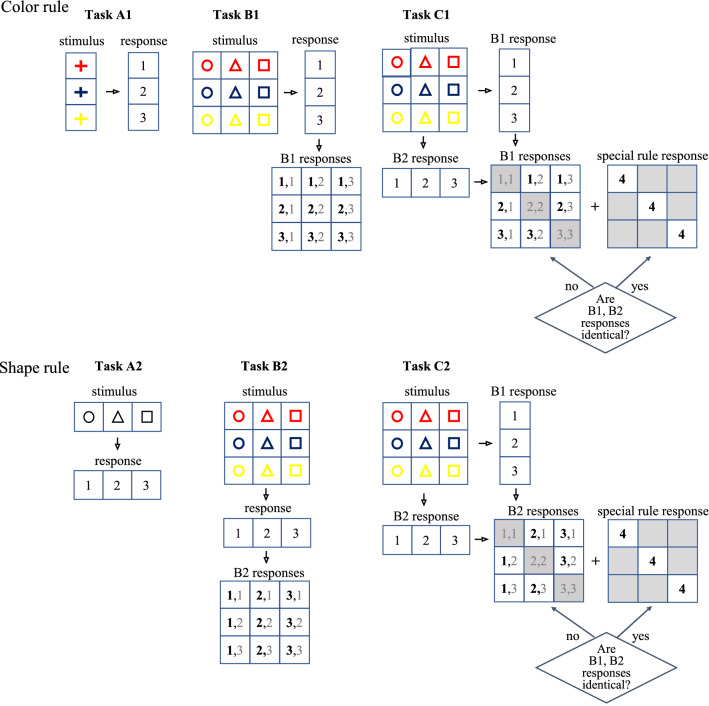
Diagrammatic representation of the task structure and cognitive operations for each rule and task. Task schemas depicting different operations of the three tasks and the relationship between the rules and responses according to the two-dimension stimuli (color and shape). In Tasks A1, B1, and C1, the main rule was keyed to color, and the response to the rule is drawn on the horizontal axis. In Tasks A2, B2, and C2, the main rule was keyed to shape, and the response to this rule is drawn on the vertical axis. Tasks A1 and A2 were direct responses to a one-dimensional stimulus. Tasks B1 and B2 were guided by the same rule as Tasks A1 and A2 but with more corresponding dimensions and stimuli. Tasks C1 and C2 comprised several steps, as follows: first, recall of each of the two rule-responses concurrently for the two-dimension stimuli; then, after evaluating these two responses, the special rule was applied if the responses were identical, otherwise the main rule was applied.

In a previous study^23^, we developed a task that demands higher-order rule selection based on evaluations of the consequence of rule-responses referring multidimensional stimulus in order to examine child cognitive development. In the current study, we used this task to investigate the neural underpinnings of rule selection behaviors. The task involved a set of two-dimensional stimuli, which were defined by color and shape. All tasks were performed using each color and shape rule separately (Fig. 1), as follows:

- In the control task (Tasks A1 and A2), participants were instructed to respond only to the one-dimensional stimulus (i.e., color or shape).
- In the rule-guided task (Tasks B1 and B2), participants were instructed to make a one-dimensional response to a two-dimensional stimulus that consisted of a combination of color and shape (i.e., they either responded to the color or the shape but not both).
- In the rule-selection task (Tasks C1 and C2), this task contains two rules: the main rule and the special rule. The main rule was to apply the same rule as the color or shape rule presented in Task B. The special rule required participants to evaluate both color and shape rules corresponding to two-dimensional stimuli and to apply the special response when special combinations occurred in the stimuli, as explained below. Participants were instructed to select one of two rules between the main rule (i.e., color or shape), applied to two-dimensional stimuli, or a special rule, based on their response evaluation. For the main rule, participants responded to one dimension of the two-dimensional stimuli (i.e., either to the color [C1] or shape [C2] as in Tasks B1 and B2). In contrast, for the special rule, participants judged whether both responses were identical (via response-button numbers) to the color and shape rules for the presented stimuli; if the response buttons were identical, participants were required to apply the special rule, whereas if they were different, participants were required to respond using the main rule.

The first two tasks required the application of one rule according to the given rules, whereas the rule-guided task required sustained awareness of both rules and more stimulus dimensions. Compared with the rule-guided task, the rule-selection task increased a number of rules but the same number of stimulus dimensions. Furthermore, the rule-selection task required adaptive rule selection based on the evaluation of the result of the two rule-responses applied to the two-dimensional stimuli. Our previous study using this task revealed marked improvement in accuracy with age, reaching adult-level performance by the age of 7 years. This age-dependent adaptive rule selection ability occurs during the same developmental period (approximately 7 years of age) when humans develop complex cognitive task processing abilities, such as those measured using the Wisconsin Card Sorting Test^24–26^. This suggests that essential and distinct neural development occurs during this period, which mediates superordinate control in cognition. Although such cognitive processes appear to be controlled by higher-order regions within the PFC that are linked with cognitive hierarchy, it is unclear which PFC region is engaged during this task because the involvement of these regions varies depending on the type of rule processing^3,27^. To understand the neural development subserving this rule processing system, it is necessary to first understand the mechanism in the mature brain and subsequently trace the neurodevelopment of superordinate control in cognition in children using the present task.

Conventional neuroimaging techniques, such as functional magnetic resonance imaging (fMRI) and positron emission tomography (PET), present certain challenges for researching children because they require strict motion restriction, and PET requires the administration of radioisotopes. It is challenging for children to keep still while performing complex tasks for a prolonged period in neuroimaging environments. In contrast, functional near-infrared spectroscopy (fNIRS) is an optical neuroimaging technique^28^ that is noninvasive and unrestrictive^29–31^. It enables real-time monitoring of cerebral hemodynamic changes associated with brain activity in children^32,33^, infants, neonates^34,35^, as well as adults^36–38^. Therefore, we used fNIRS to identify the brain regions that are engaged during the performance of the above-mentioned tasks in adults.

## Results

### Behavioral data

Table 1 shows the means and standard deviations of all tasks. Behavioral data analyzed using repeated-measures analysis of variance (ANOVA) for the factor of task revealed significant main effects of accuracy (*F*(1.22, 47.43) = 25.98, *p* < 0.0001, η_p_^2^ = 0.382) and reaction time (RT) (*F*(1.20, 50.05) = 560.29, *p* < 0.0001, η_p_^2^ = 0.930). Post hoc comparisons of accuracy among the three conditions (i.e., Tasks A, B, and C) showed significantly lower accuracy in Task C than in Tasks A (mean difference (MD) (%)= − 3.02, standard error (SE) = 0.59, *p* < 0.0001) and B (MD (%)= − 2.97, SE = 0.57, *p* < 0.0001). There was no significant difference between Tasks A and B (MD (%)= 0.04, SE = 0.17, *p* = 1). A significantly longer RT was observed in Task C than in both Tasks A (MD (ms)= 486.78, SE = 17.39, *p* < 0.0001) and B (MD (ms)= 415.94, SE = 19.71, *p* < 0.0001). In addition, the RT in Task B was significantly longer than in Task A (MD (ms)= 70.83, SE = 7.05, *p* < 0.0001).

**Table 1:** Accuracy and response times.

	Mean response time (ms)	Mean accuracy (%)
Task A	570.07 (103.12)	99.66 (0.83)
Task B	649.90 (117.61)	99.61 (0.86)
Task C	1965.85 (154.62)	96.64 (3.78)

Values in parentheses are standard deviations.

#### Changes in oxygenated hemoglobin (oxy-Hb)

To determine the difference in oxy-Hb responses between the primary tasks, the mean integral values of the Task B and C conditions were subtracted from the control conditions (Task A; i.e., ∆oxy-Hb_B-A_, and ∆oxy-Hb_C-A_, respectively). A two-factorial repeated measures ANOVA for changes in oxy-Hb revealed significant main effects of task (∆oxy-Hb_B-A_ and ∆oxy-Hb_C-A_; *F*(1, 42 = 21.11), *p* < 0.001, η_p_^2^ = 0.324) and cortical region (*F*(2.56, 107.42) = 5.878, *p* = 0.002, η_p_^2^ = 0.123). Furthermore, there was a significant interaction between condition and cortical region (F(3.529, 148.21) = 3.002, *p* = 0.025, η_p_^2^ = 0.067).

**Figure 2:**
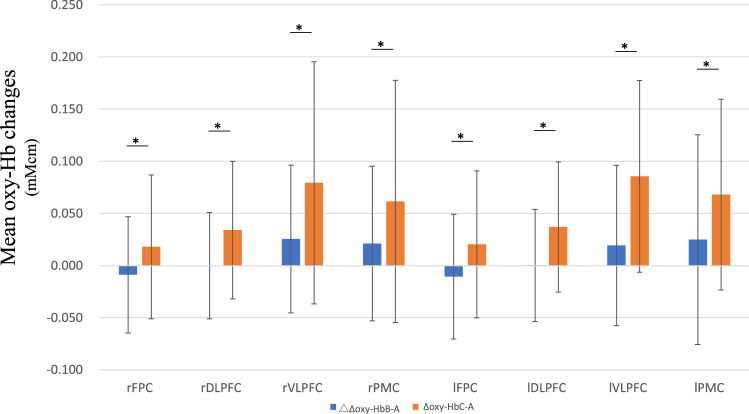
Mean oxygenated hemoglobin (oxy-Hb) signal changes between tasks (∆oxy-Hb_B–A_ and ∆oxy-Hb_C–A_) in each region of interest. Error bars indicate standard deviations. *p < 0.05.

Because we observed a significant interaction, we performed post hoc comparisons to analyze the effect of the tasks on each cortical region. A significant difference was observed between tasks in all regions of interest (ROIs): the right FPC (MD (mM cm)= 0.03, SE = 0.01, *p* = 0.0081), the right DLPFC (MD (mM cm)= 0.03, SE = 0.01, *p* = 0.0016), the right VLPFC (MD (mM cm)= 0.05, SE = 0.01, *p* = 0.001), the right PMC (MD (mM cm)= 0.04, SE = 0.02, *p* = 0.009), the left FPC (MD (mM cm)= 0.03, SE = 0.01, *p* = 0.0053), the left DLPFC (MD (mM cm)= 0.04, SE = 0.01, *p* = 0.0011), the left VLPFC (MD (mM cm)= 0.07, SE = 0.01, *p* < 0.001), and the left PMC (MD (mM cm)= 0.04, SE = 0.01, *p* = 0.0021) at a threshold of *p* < 0.05 (Fig. 2). Thus, we observed a significantly higher increase in the levels of oxy-Hb in ∆oxy-Hb_C–A_ in all regions of the PFC and the PMC than in those in ∆oxy-Hb_B–A_. These results indicated that broader regions of the PFC and PMC are involved in the rule-selection mechanism activated during Task C.

## Discussion

We used fNIRS and a novel task to investigate the neural underpinnings of the processing of adaptive rule selection, which was contingent on an evaluation of the response. We found that a broader region of the bilateral PFC and the PMC was activated during the task in which rule selection required evaluation of the consequence of a rule-response (Task C) than during the task that demanded a rule-guided response (Task B). Our results suggested that a broad range of prefrontal regions and the PMC are involved in the superordinate control of adaptive rule selection behaviors that demand multiple cognitive operations.

Previous studies have elucidated the neural underpinnings of rule-guided cognitive control in the PFC. Specifically, it has been suggested that according to gradient theories, the FPC functions as the apex of the cognitive hierarchy within the PFC and is involved in the representation of the highest level of abstract rules, the selection of goal-relevant information^8–10,16^, the complexity of rule-stimulus relationships^39–41^, and the integration of the outcomes of multiple cognitive processes involving relational operations^42^. It has been suggested that the PFC is engaged when problems involve more than one discrete cognitive process and when the application of the results of two or more separate cognitive operations is required to fulfill a higher behavioral goal^42^. In contrast, within the lateral PFC, the highest level of cognitive control has been proposed to be exerted within the mid-lateral region along the dorsal–ventral axis rather than within the rostral PFC^18–21^. Indeed, it has been demonstrated that the rostral-to-caudal processing hierarchy is dissociated into a ventral and a dorsal component and peaks in the mid-DLPFC, which exerts the highest level of cognitive control^21,43^. The DLPFC is thought to play an integrative role to coordinate stimulus- and context-related information with temporal information about the task^21^. Considering these views, we predicted that either the FPC or DLPFC would play a dominant role as the apex of the frontal control hierarchy during Task C, which demands higher level control for multiple cognitive operations. However, our results showed that neither the FPC nor DLPFC showed dominant activation, although both regions were involved in rule selection processing in Task C. This indicated that the implementation of these multiple cognitive operations does not rely solely on higher-order domain-specific processing but rather, the broader activation of the lateral PFC induced by task C, seemingly because of the presence of another cognitive control system to coordinate multiple cognitive operations in parallel. Alternatively, the broader activation may have been attributed to the specific design of our task.

Cognitive processing in Task C requires, at minimum, the following processes: (A) memory processing to concurrently recall two rule-responses according to stimuli, (B) response evaluation to judge these responses between the button-numbers, (C) rule selection based on the result of response evaluation, and (D) action choice for which button to press to follow the rule-instruction. We hypothesized that there would be a specific region within the PFC that is specifically responsible for coordinating the multiple cognitive operations of Task C. However, we did not find specific activation in the lateral PFC. Nevertheless, although some previous studies have supported the existence of a functional gradient, other studies have found a lower degree of specialization within the PFC. Previous fMRI studies have sought to determine whether rules at different hierarchical levels are either represented by distinct locations in the PFC or encoded by a single system^3^. Results have revealed no major spatial differences between region encoding rules of different hierarchical levels, which suggests that the brain represents conditional rules similarly regardless of their level in the hierarchy. Thus, human control systems do not organize task representation according to this dimension^3^. Rather, such multiple cognitive operations may be subserved by a large-scale brain network termed the multiple-demand (MD) network that flexibly organizes and controls cognitive manipulations across diverse mental activities^44,45^. Indeed, many studies have shown that the same regions are coactivated when subjects are engaged in various types of cognitive tasks^46–50^. In particular, the two control network regions as a dual-network architecture, the lateral frontoparietal (FP) and cingulo-opercular (CO) cortices, are thought to be responsible for cognitive control at different timescales: the FP control network is preferentially engaged in the initiation and rapid adaptive cognitive control of shorter-term processes, whereas the CO control network provides sustained task-set maintenance of longer-term processes^51–54^. Specifically, the FP cortex provides cognitive control over externally- versus internally-generated information^55^, is active during a range of cognitive tasks, and plays a key role in goal-driven activities^44,52^. It has also been suggested that the FP network contributes to stronger deliberate control via the maintenance and implementation of complex task rules, which is a process that involves the DLPFC or rostrolateral PFC^56^. In addition, there is compelling evidence of functional specialization within the MD region, whereby certain regions respond more than others to specific manipulations^57^. Given the results of previous studies and our findings, it is reasonable to assume that the FP network is involved in the manipulation of multiple cognitive demands induced by task C, while the DLPFC and FPC exert their respective functions within their networks.

Furthermore, it is possible that the specific design of our control task (i.e., Task B) contrasted against Task C may have partly contributed to the widespread activation observed. In our task, Task B required participants to recall a single rule in response to a single dimension of a two-dimensional stimulus, whereas Task C required participants to recall two rules in response to two-dimensional stimuli together; moreover, an additional special rule needed to be recalled if necessary. Thus, there was a clear gap in the manipulation of memory load between Tasks B and C, which may have contributed to broader PFC activation during Task C because an increasing memory load has been shown to augment the overall activity in brain regions such as the prefrontal, parietal, and sensory cortices^58,59^. However, it should be noted that broader PFC activation in Task C alone may not account for the increased memory load, which may also result from cognitive operations that manipulate these memories and guide responses. Indeed, the increase in activity may not have been induced simply by an increase in memory load, especially within the PFC, because such an activity increase is only observed in areas that overlap with the control task, regardless of the memory load of the task^46,47^. Thus, our finding that the PFC is activated to a broader extent in Task C than in Task B is likely due to diverse cognitive demands^44,50^. Further research is needed that includes additional control tasks for the rule-induction task to control memory load and elucidate the network regions relevant to the rule-selection task.

## Conclusion

The present study in adult participants investigated the neural underpinnings of a novel task (Task C) designed to assess adaptive rule selection behavior based on the evaluation of the consequences of a given rule-response. We hypothesized that specific regions within the lateral PFC would be responsible for the task process as a superordinate control system that organizes the rule-selection behavior involved in multiple cognitive operations based on the hierarchical model. Results showed that the FPC and DLPFC contributed to the execution of Task C, but their activity was non-specific; moreover, a broader range of regions in the lateral PFC and PMC were engaged during the execution of this task. We speculated that this activation pattern was supported by a large brain network centered on the FP control network. Because our previous findings revealed that the emergence and formation of this rule-control ability are age-dependent and established by the age of 7 years^23^, we determined that this task could be used to examine the neural developmental underpinnings of the FP control network. In future studies, we plan to further improve the current task protocol and apply the task to children to explore the neurodevelopment of adaptive rule selection based on the control of multiple cognitive operations. It is worth highlighting that our task can be performed by participants as young as preschool age; moreover, the imaging method is particularly useful for studying children. Our protocol will likely contribute to the future understanding of the developmental process of higher-level cognitive control in humans.

## Methods

### Participants

Participants were recruited from the University of Shizuoka, the Hamamatsu University School of Medicine, and the City of Hamamatsu via local advertisements. The study protocol was approved by the Review Committee of Hamamatsu University School of Medicine (16-146) and was conducted in accordance with the Declaration of Helsinki. We obtained written informed consent from all participants prior to initiating data collection. Each participant was paid 7000 yen for their participation.

Forty-seven healthy Japanese adults participated in our study (mean age 22 years, standard deviation [SD] 3 years, range 18–32 years; 19 women). Inclusion criteria included normal or corrected-to-normal vision, no self-reported neurological or psychiatric history, and no anatomical brain abnormalities. During the first visit, we collected demographic information and administered several scales: the Wechsler Adult Intelligence Scale^60^ to assess Intelligence Quotient (IQ), the Autism-Spectrum Quotient Scale^61^ to establish the presence of autism spectrum disorder, and Conner’s Adult attention-deficit/hyperactivity disorder (ADHD) Rating Scale (CAARS)^62^ to establish the presence of ADHD. One female participant was excluded because of a full-scale IQ lower than 80, and three participants (2 men and 1 woman) were excluded because of a high cut-off value (T-score) for determining clinical significance (> 65) on the CAARS, as per our criterion for ADHD symptoms. The final participants (N = 43 participants; age range 18–32 years) included 19 women with a mean age of 21.8 years (SD 3.7 years) and 24 men with a mean age of 23.1 years (SD 2.7 years). Both right- and left-handed individuals were included (40 right-handed with a score of > 40 on the Edinburgh Handedness Inventory^63^ and three left-handed with a score of < 40 on the Edinburgh Handedness Inventory). The fNIRS measurement was performed during the second visit.

### Task design

The experiment was conducted using the Presentation^®^ software (Neurobehavioral Systems Inc, Albany, CA, USA) on a Dell computer. Stimuli were presented on a 17-inch computer monitor at a refresh rate of 60 Hz. Participants were seated in a comfortable chair in a dim chamber at a distance of approximately 50 cm from the screen. They were instructed to stay relaxed and to respond by pressing one of the three or four pre-specified keys on a button box (10 × 14 × 3 cm) using their dominant hand.

The task, which is described in detail above, demands a unique rule processing approach for responding to two dimensions of stimuli (i.e., color and shape; Fig. 1). We set three tasks (Tasks A, B, and C), and all tasks contained two rules (color [“1”] and shape [“2”]), which were executed separately. The control task (Task A) used one rule that required a response to one dimension of the stimulus. The rule-guided task (Task B) used one rule that required a response to one dimension of a two-dimensional stimulus. The rule-selection task (Task C) required the selection of either the main rule (the same rule used in Task B) or a special rule based on the evaluation of responses to both the color and shape rules (Fig. 1). All tasks imposed two color and shape rules as follows. The color rule demanded the use of numbered buttons (red = button 1, blue = button 2, and yellow = button 3) in response to three possible color stimuli. The shape rule demanded the use of numbered buttons (circle = button 1, triangle = button 2, and square = button 3) in response to three possible shape stimuli. In Task C, the main rule demanded the same response as in Task B, with corresponding numbered buttons in response to the color (Task C1) or shape (Task C2) of the stimuli. However, an additional rule was included that demanded a response (button 4) based on the color and shape rules and the selection of a rule to follow (Fig. 1). The task order was randomly assigned, but the governing rule (color or shape response) was maintained (i.e., the two rules in each task were not sequentially executed [e.g., color rule: Task B → Task A → Task C, followed by the shape rule: Task A → Task C → Task B). This minimized the influence of conflict interference between the color and shape rules that could be induced by switching between rules. In addition, as our analysis design compared Tasks B and C, both of which contained conflict, the contrast between these two tasks contributed to further controlling for possible conflict effects. Furthermore, the order of implementation of these rules (i.e., color-shape and shape-color) was counterbalanced across participants.

The behavioral performance, accuracy (%), and RTs were calculated individually by averaging the performance of the two rules (color and shape) to control for the influence of trial order (first and second) and rule dimensions (color and shape).

#### Sequence of stimuli and temporal structure within each block

**Figure 3:**
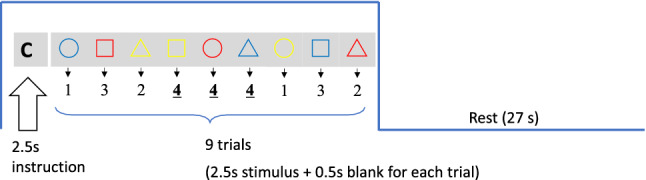
Task sequences. For all three tasks, there were six task blocks. Each task block (29.5 s) consisting of nine trials was followed by a rest period (27 s).

For all tasks, each trial consisted of an instruction cue, a blank screen, and a target stimulus. The instruction cue (“Start!”) was presented for 2.5 s, and subjects were informed about which of three tasks would be assigned (Task A, B, or C). A target stimulus was then presented for 2 s, followed by a blank screen for 0.5 s. Each task block (29.5 s) consisted of nine trials and was followed by a rest period (27 s; Fig. 3). In each of the three task conditions, there were six task blocks and a total of 54 trials. The tasks were presented in a pseudorandomized order. The experiment consisted of a continuous series of three separate conditions (i.e., Task A, B, or C), with each run performed separately as a color-rule phase or a shape-rule phase, as described above.

Before the fNIRS measurement, each subject underwent a practice phase comprising 27 trials. The experimental phase began once the subject achieved an accuracy rate of 85%, which was achieved by all subjects within three blocks.

### fNIRS data acquisition

**Figure 4:**
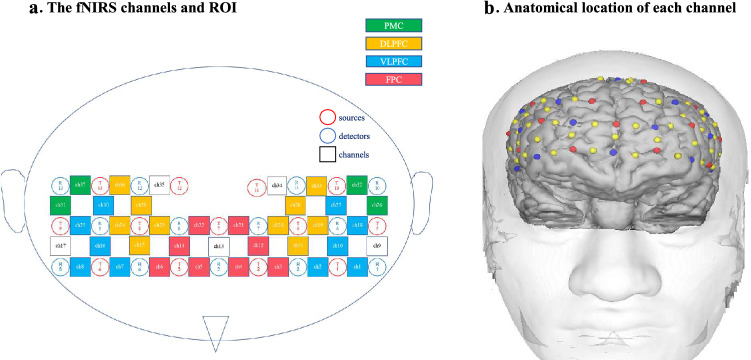
Schematics of the optode locations. (**a**) The functional near-infrared spectroscopy (fNIRS) channels and the regions of interest (ROIs). A total of 26 optodes, including 13 sources and 13 detectors, were arranged on the scalp to enable the measurement of 37 channels (Ch). The ROIs of the fNIRS Ch comprised areas of the ventrolateral prefrontal cortex (VLPFC; blue: Ch 7–8, 16, 25, and 30 in the right hemisphere; Ch 1–2, 10, 18, and 27 in the left hemisphere), dorsolateral prefrontal cortex (DLPFC; yellow: Ch 15, 23–24, 29, and 36 in the right hemisphere; Ch 11, 19–20, 28, and 33 in the left hemisphere), frontopolar cortex (FPC; pink: Ch 5–6, 14, and 22 in the right hemisphere; Ch 3–4, 12, and 21 in the left hemisphere), and the premotor cortex (PMC; green: Ch 31 and 37 in the right hemisphere; Ch 22 and 26 in the left hemisphere). The numbers (Ch 1–37) indicate the channels of the fNIRS probe. (**b**) Anatomical location of each channel. The anatomical locations of the optodes were digitized and superimposed onto three-dimensional surface-rendered magnetic resonance images of the brain.

Hemodynamic responses during stimuli presentation were recorded using a multichannel fNIRS imaging system (FOIRE3000, Shimadzu Co., Kyoto, Japan) at three wavelengths (780, 805, and 830 nm), and changes in Hb were calculated according to the modified Beer–Lambert law^64^. Thirteen pairs of illuminating and detecting light guides were arranged on the forehead in pairs, with a fixed spacing of 3.0 cm to create a custom 37-channel (Ch) probe (Fig. 4a). Sampling intervals were set to 130 ms. The accuracy and utility of this system have been verified previously^65^.

For spatial registration, the probe-set was placed on the head according to the relevant standard positions of the international 10–20 system for electroencephalography (EEG) electrode placement^66^. A probe holder was placed using Cz and Fpz, and the left probe holder was placed so that F7 corresponded to the region between Ch 1 and Ch 2 (the location of T1 approximately corresponded to F7). Likewise, the right probe holder was placed symmetrically so that F8 corresponded to the region between Ch 7 and Ch 8 (the location of T4 approximately corresponded to F8). The neuroanatomical locations beneath each light guide, which were located using the international 10–20 system for EEG, were confirmed in six participants who underwent MRI. An electromagnetic tracking device (FASTRAK, Polhemus, USA) was used to digitize the light guide positions, which were marked on the head surface and registered to three-dimensional surface-rendered magnetic resonance images of the brain. Additionally, Montreal Neurological Institute coordinates for the channels were obtained using NIRS-SPM^67^ in MATLAB R2021b (Mathworks, Natick, MA), and the corresponding anatomical locations of each channel were determined (Fig. 4a).

**Figure 5:**
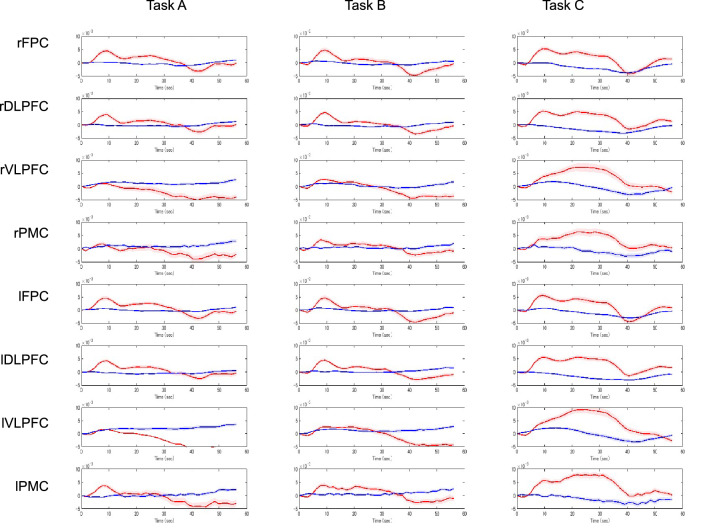
The averaged waveforms of oxygenated (oxy-Hb) and deoxygenated hemoglobin (deoxy-Hb) of each task for all regions of interest (ROIs). The red lines indicate the observed timelines of the oxy-Hb signal, and the blue lines indicate the deoxy-Hb signal. The first 29.5 s indicate the task block period, and the second half indicates the rest period.

The data of each channel were then clustered into anatomical regions according to shared anatomy, and these groupings were used for the task-related activation analyses. Of the 37 channels measured, those located outside the PFC (i.e., Ch 9 and Ch 17) and those that were the sole channel within a region (i.e., Ch 13, Ch 34, and Ch 35) were excluded. The average number of channels in each region was 3.5 ± 1.0. Grouping was achieved by automatically identifying four bilateral ROIs from the acquired channels: (i) the VLPFC (Brodmann’s areas [BAs] 45/47 and inferior frontal gyrus), (ii) the DLPFC (BAs 9/46 and the middle frontal gyrus), (iii) the FPC (BA10), and (iv) the PMC (BA6; Fig. 4b). The time courses of oxy-Hb and deoxy-Hb from the grand-averaged data observed in the 43 participants for all ROIs are provided in Fig. 5.

### fNIRS data processing

For statistical analyses, the fNIRS data were analyzed using Matlab (Version 2017a, Mathworks Inc., Natick, MA, USA). We focused on oxy-Hb because it is more sensitive to changes in cerebral blood flow^68–70^, has better signal-to-noise ratio^70^, and has higher retest reliability^71^ than deoxy-Hb and total-Hb signals. In addition, deoxy-Hb signals are considered unreliable because of their low-signal intensities and non-negligible crosstalk errors^72^. Furthermore, because previous fNIRS studies have exclusively examined the oxy-Hb signal, we also used the oxy-Hb signal as our primary measure. We performed drift correction between the first data point and the endpoint in the time-continuous fNIRS data before extracting the task blocks. We excluded blocks and channels with poor signal conditions, such as those affected by excessive high-frequency noise, representing insufficient optical signals, and abrupt simultaneous changes in oxy-Hb and deoxy-Hb signals, indicative of body movement. Exclusions were made only after establishing inter-rater agreement based on independent visual examinations. For each task block, baseline correction was performed by assigning a value of zero to the signal data at the onset of each block. After discarding blocks with artifacts, we calculated an integral value of the oxy-Hb signal during each task block (19.5 s from 10 s after the presentation of the instructional stimulus), and the values were then averaged across the blocks (six blocks) of each task for each subject. The mean integral value of each task was further calculated as Task B–Task A (∆oxy-Hb_B-A_) and Task C–Task A (∆oxy-Hb_C-A_), rather than comparing the conditions to a resting baseline because comparing a task to a resting baseline can result in oxygenation differences that are associated with the execution of a challenging task instead of the construct of interest^73^. Moreover, this minimized the effect of artifacts due to skin blood flow.

### Statistical analysis

All statistical analyses were carried out using the SPSS statistical package ver. 25 (SPSS Inc, Chicago, IL, USA). Task performance (accuracy [%] and RT) was analyzed using repeated-measures ANOVA to compare the three tasks (i.e., Tasks A, B, and C). Two-factorial repeated-measures ANOVA was used to examine the effect of the response magnitude of oxy-Hb changes on the ROIs (bilateral VLPFC, DLPFC, FPC, and PMC; within-participant factors) and tasks (∆oxy-Hb_B–A_ and ∆oxy-Hb_C–A_; repeated measures). If a significant interaction was observed between cortical regions and tasks, we performed post hoc analyses using Bonferroni correction to analyze the effect of the tasks on each ROI. Statistical significance was set to *p* < 0.05.

## References

[1] Bouchacourt F, Palminteri S, Koechlin E, Ostojic S (2020). Temporal chunking as a mechanism for unsupervised learning of task-sets. Elife.

[2] Wallis JD, Miller EK (2003). From rule to response: Neuronal processes in the premotor and prefrontal cortex. J. Neurophysiol..

[3] Pischedda D, Gorgen K, Haynes JD, Reverberi C (2017). Neural representations of hierarchical rule sets: The human control system represents rules irrespective of the hierarchical level to which they belong. J. Neurosci..

[4] Monchi O, Petrides M, Petre V, Worsley K, Dagher A (2001). Wisconsin Card Sorting revisited: Distinct neural circuits participating in different stages of the task identified by event-related functional magnetic resonance imaging. J. Neurosci..

[5] Wallis JD, Anderson KC, Miller EK (2001). Single neurons in prefrontal cortex encode abstract rules. Nature.

[6] Reverberi C, Gorgen K, Haynes JD (2012). Distributed representations of rule identity and rule order in human frontal cortex and striatum. J. Neurosci..

[7] Reverberi C, Gorgen K, Haynes JD (2012). Compositionality of rule representations in human prefrontal cortex. Cereb. Cortex.

[8] Badre D, D'Esposito M (2009). Is the rostro-caudal axis of the frontal lobe hierarchical?. Nat. Rev. Neurosci..

[9] Koechlin E, Ody C, Kouneiher F (2003). The architecture of cognitive control in the human prefrontal cortex. Science.

[10] Koechlin E, Summerfield C (2007). An information theoretical approach to prefrontal executive function. Trends Cogn. Sci..

[11] Badre D (2008). Cognitive control, hierarchy, and the rostro-caudal organization of the frontal lobes. Trends Cogn. Sci..

[12] Fuster JM (2000). Executive frontal functions. Exp. Brain Res..

[13] Botvinick MM (2008). Hierarchical models of behavior and prefrontal function. Trends Cogn. Sci..

[14] Christoff K, Keramatian K, Gordon AM, Smith R, Madler B (2009). Prefrontal organization of cognitive control according to levels of abstraction. Brain Res..

[15] O'Reilly RC (2010). The what and how of prefrontal cortical organization. Trends Neurosci..

[16] Badre D, D'Esposito M (2007). Functional magnetic resonance imaging evidence for a hierarchical organization of the prefrontal cortex. J. Cogn. Neurosci..

[17] Nee DE, Brown JW (2012). Rostral-caudal gradients of abstraction revealed by multi-variate pattern analysis of working memory. Neuroimage.

[18] Margulies DS (2016). Situating the default-mode network along a principal gradient of macroscale cortical organization. Proc. Natl. Acad. Sci. U. S. A..

[19] Nee DE, D'Esposito M (2016). The hierarchical organization of the lateral prefrontal cortex. Elife.

[20] Badre D, Nee DE (2018). Frontal cortex and the hierarchical control of behavior. Trends Cogn. Sci..

[21] Schumacher FK, Schumacher LV, Schelter BO, Kaller CP (2019). Functionally dissociating ventro-dorsal components within the rostro-caudal hierarchical organization of the human prefrontal cortex. Neuroimage.

[22] Alexander WH, Brown JW (2015). Hierarchical error representation: A computational model of anterior cingulate and dorsolateral prefrontal cortex. Neural Comput..

[23] Harada T, Tsuruno M, Shirokawa T (2018). Developmental trajectory of rule management system in children. Sci. Rep..

[24] Miyake A (2000). The unity and diversity of executive functions and their contributions to complex "frontal lobe" tasks: A latent variable analysis. Cogn. Psychol..

[25] Huizinga M, Dolan CV, van der Molen MW (2006). Age-related change in executive function: Developmental trends and a latent variable analysis. Neuropsychologia.

[26] Lehto J, Juujärvi P, Kooistra L, Pulkkinen L (2003). Dimensions of executive functioning: Evidence from children. Br. J. Dev. Psychol..

[27] Jeon HA (2014). Hierarchical processing in the prefrontal cortex in a variety of cognitive domains. Front. Syst. Neurosci..

[28] Wilcox T, Biondi M (2015). fNIRS in the developmental sciences. Wiley Interdiscip. Rev. Cogn. Sci..

[29] Watanabe E (1998). Non-invasive assessment of language dominance with near-infrared spectroscopic mapping. Neurosci. Lett..

[30] Ikegami T, Taga G (2008). Decrease in cortical activation during learning of a multi-joint discrete motor task. Exp. Brain Res..

[31] Hoshi Y (2007). Functional near-infrared spectroscopy: Current status and future prospects. J. Biomed. Opt..

[32] Hoshi Y, Chen SJ (2002). Regional cerebral blood flow changes associated with emotions in children. Pediatr. Neurol..

[33] Sugiura L (2011). Sound to language: Different cortical processing for first and second languages in elementary school children as revealed by a large-scale study using fNIRS. Cereb. Cortex.

[34] Taga G, Asakawa K, Maki A, Konishi Y, Koizumi H (2003). Brain imaging in awake infants by near-infrared optical topography. Proc. Natl. Acad. Sci. U. S. A..

[35] Aslin RN, Shukla M, Emberson LL (2015). Hemodynamic correlates of cognition in human infants. Annu. Rev. Psychol..

[36] Maki A (1995). Spatial and temporal analysis of human motor activity using noninvasive NIR topography. Med. Phys..

[37] Holmes E (2019). Cognitive enhancement by transcranial photobiomodulation is associated with cerebrovascular oxygenation of the prefrontal cortex. Front. Neurosci..

[38] Hoshi Y (2003). Spatiotemporal characteristics of hemodynamic changes in the human lateral prefrontal cortex during working memory tasks. Neuroimage.

[39] Christoff K, Gabrieli JDE (2000). The frontopolar cortex and human cognition: Evidence for a rostrocaudal hierarchical organization within the human prefrontal cortex. Psychobiology.

[40] Christoff, K., Keramatian, K. *Abstraction of Mental Representations: Theoretical Considerations and Neuroscientific Evidence.* 107–126 (Oxford University Press, 2007).

[41] Christoff K, Ream JM, Geddes LP, Gabrieli JD (2003). Evaluating self-generated information: Anterior prefrontal contributions to human cognition. Behav. Neurosci..

[42] Ramnani N, Owen AM (2004). Anterior prefrontal cortex: Insights into function from anatomy and neuroimaging. Nat. Rev. Neurosci..

[43] Bahlmann J, Blumenfeld RS, D'Esposito M (2015). The rostro-caudal axis of frontal cortex is sensitive to the domain of stimulus information. Cereb. Cortex.

[44] Duncan J (2010). The multiple-demand (MD) system of the primate brain: Mental programs for intelligent behaviour. Trends Cogn. Sci..

[45] Duncan J, Assem M, Shashidhara S (2020). Integrated intelligence from distributed brain activity. Trends Cogn. Sci..

[46] Klingberg T (1998). Concurrent performance of two working memory tasks: Potential mechanisms of interference. Cereb. Cortex.

[47] Adcock RA, Constable RT, Gore JC, Goldman-Rakic PS (2000). Functional neuroanatomy of executive processes involved in dual-task performance. Proc. Natl. Acad. Sci. U. S. A..

[48] Cabeza R, Nyberg L (2000). Imaging cognition II: An empirical review of 275 PET and fMRI studies. J. Cogn. Neurosci..

[49] Cole MW, Schneider W (2007). The cognitive control network: Integrated cortical regions with dissociable functions. Neuroimage.

[50] Fedorenko E, Duncan J, Kanwisher N (2013). Broad domain generality in focal regions of frontal and parietal cortex. Proc. Natl. Acad. Sci. U.S.A..

[51] Dosenbach NUF (2006). A core system for the implementation of task sets. Neuron.

[52] Dosenbach NUF (2007). Distinct brain networks for adaptive and stable task control in humans. Proc. Natl. Acad. Sci. U.S.A..

[53] Dosenbach NUF, Fair DA, Cohen AL, Schlaggar BL, Petersen SE (2008). A dual-networks architecture of top-down control. Trends Cogn. Sci..

[54] Crittenden BM, Mitchell DJ, Duncan J (2016). Task encoding across the multiple demand cortex is consistent with a frontoparietal and cingulo-opercular dual networks distinction. J. Neurosci..

[55] Spreng RN, Stevens WD, Chamberlain JP, Gilmore AW, Schacter DL (2010). Default network activity, coupled with the frontoparietal control network, supports goal-directed cognition. Neuroimage.

[56] Zamani A, Carhart-Harris R, Christoff K (2022). Prefrontal contributions to the stability and variability of thought and conscious experience. Neuropsychopharmacology.

[57] Shenhav A, Botvinick MM, Cohen JD (2013). The expected value of control: An integrative theory of anterior cingulate cortex function. Neuron.

[58] Curtis CE, D'Esposito M (2003). Persistent activity in the prefrontal cortex during working memory. Trends Cogn. Sci..

[59] Ma WJ, Husain M, Bays PM (2014). Changing concepts of working memory. Nat. Neurosci..

[60] Wechsler, D. *Wechsler Adult Intelligence Scale*. 3rd ed. (The Psychological Corporation, 1997).

[61] Baron-Cohen S, Wheelwright S, Skinner R, Martin J, Clubley E (2001). The autism-spectrum quotient (AQ): Evidence from Asperger syndrome/high-functioning autism, males and females, scientists and mathematicians. J. Autism Dev. Disord..

[62] Macey KD (2003). Conners' adult ADHD rating scales (CAARS). Arch. Clin. Neuropsychol..

[63] Oldfield RC (1971). The assessment and analysis of handedness: The Edinburgh inventory. Neuropsychologia.

[64] Delpy DT (1988). Estimation of optical pathlength through tissue from direct time of flight measurement. Phys. Med. Biol..

[65] Kohno S, Hoshi Y (2016). Spatial distributions of hemoglobin signals from superficial layers in the forehead during a verbal-fluency task. J. Biomed. Opt..

[66] Okamoto M (2004). Three-dimensional probabilistic anatomical cranio-cerebral correlation via the international 10–20 system oriented for transcranial functional brain mapping. Neuroimage.

[67] Ye JC, Tak S, Jang KE, Jung J, Jang J (2009). NIRS-SPM: Statistical parametric mapping for near-infrared spectroscopy. Neuroimage.

[68] Hoshi Y, Kobayashi N, Tamura M (2001). Interpretation of near-infrared spectroscopy signals: A study with a newly developed perfused rat brain model. J. Appl. Physiol..

[69] Hoshi Y (2003). Functional near-infrared optical imaging: Utility and limitations in human brain mapping. Psychophysiology.

[70] Strangman G, Culver JP, Thompson JH, Boas DA (2002). A quantitative comparison of simultaneous BOLD fMRI and NIRS recordings during functional brain activation. Neuroimage.

[71] Plichta MM (2006). Event-related functional near-infrared spectroscopy (fNIRS): Are the measurements reliable?. Neuroimage.

[72] Boas DA, Dale AM, Franceschini MA (2004). Diffuse optical imaging of brain activation: Approaches to optimizing image sensitivity, resolution, and accuracy. Neuroimage.

[73] Aslin RN (2012). Questioning the questions that have been asked about the infant brain using near-infrared spectroscopy. Cogn. Neuropsychol..

